# Arterial Thrombus in a Protein C Deficient Patient

**DOI:** 10.7759/cureus.6130

**Published:** 2019-11-12

**Authors:** Eyad Mansour, Alena Isaeva

**Affiliations:** 1 Cardiology, Lebanese Geitaoui Hospital, Beirut, LBN

**Keywords:** protein c deficiency, arterial thrombus, acute myocardial infarction

## Abstract

Protein C is a Vitamin K derivative that plays an essential role in anticoagulation. Protein C deactivates clotting factors Va and VIIIa; therefore, a deficiency in this protein leads to over expression and activation of these factors and essentially a hypercoagulable, prothrombotic state. Although studies have shown that the cardinal manifestation of protein C deficiency is venous thromboembolism, we present a case of a patient in his third decade with a myocardial infarction on a background of protein C deficiency and minimal cardiovascular risk factors. Similar cases of patients presenting with arterial thrombus on background of protein C deficiency have been reported; therefore, it is imperative to acknowledge protein C deficiency as a possible cause of acute, premature myocardial infarctions in young patients with minimal or no risk for cardiovascular disease.

## Introduction

Protein C, also known as coagulation factor XIV [1] is a Vitamin K dependent zymogen. The inactive zymogen circulates in the blood at a concentration of 4 μg/mL and is catalyzed into its active serine-protease enzyme activated protein C form by thrombin when bound to endothelial glycoprotein thrombomodulin [2-3]. The activated form of Vitamin C plays a crucial role in regulating anticoagulation, inflammation, preserving permeability of blood vessels, and cell death. Its anticoagulation function is achieved through the inactivation of clotting factors Va and VIIIa which are essential for factor X activation and the generation of thrombin [4]. Consequently, a deficiency in protein C induces hypercoagulability and thrombophilia. The most common manifestation of a protein C deficiency is venous thromboembolism; however, there has been several reports of arterial disease and myocardial infarctions occurring in young adults with congenital protein C deficiency but no significant cardiovascular risk factors [5]. An association between protein C deficiency and arterial thrombosis is controversial and a correlation between the two has not yet been made. Here we report a case of a 32-year-old patient who presented with a myocardial infarction secondary to a protein C deficiency and some minor cardiovascular risk factors. 

## Case presentation

**Figure 1:**
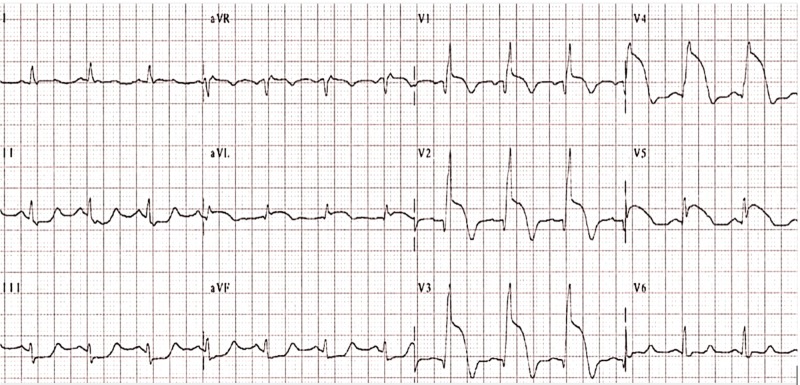
Electrocardiograph showing anteroseptal ST elevation.

**Figure 2:**
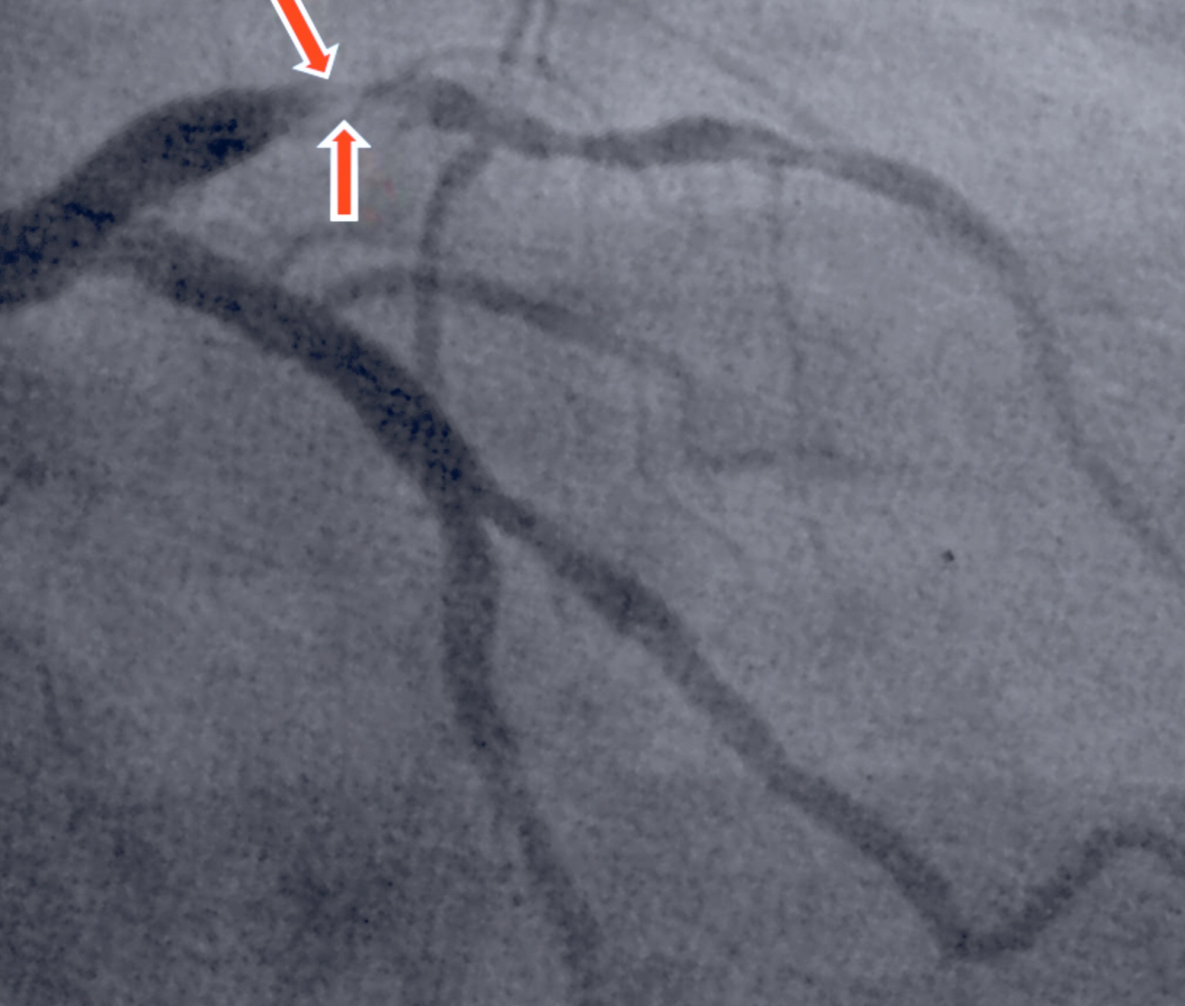
Angiogram revealed total thrombotic occlusion of the left anterior descending artery.

A 32-year-old Middle Eastern male was admitted to the hospital following a 2-h episode of severe retrosternal chest pain that radiated to his left shoulder. Shortness of breath, diaphoresis, and nausea accompanied the chest pain. His medical history was significant for alcohol and tobacco use (two pack years), and a negative family history of cardiovascular disease. The patient has no other major cardiovascular risk factors. The patient denied any abuse of cocaine or illicit drugs. Physical examination was unremarkable and hemodynamics was stable at admission. Immediately after his arrival the patient was admitted to the ED where electrocardiography showed ST elevations maximal in V1-V4 and ST depression in leads II, III, and arteriovenous fistula (AVF) (Figure 1). Troponin T concentration was 1.10 ng/mL (normal 0.0-0.10 ng/mL) and creatine kinase peaked at 14375 U/L. Coronary angiography was then preformed and revealed total occlusion of the left anterior descending artery (LAD) (Figure 2). The remaining coronary arteries were normal. Percutaneous coronary intervention was preformed and a stent was placed as part of the immediate management of anterior wall infarction. Coagulation profile was then assessed and a diagnosis of protein C deficiency was made (Table 1). Following admission and adequate management, the patient’s chest pain resolved although serial cardiac enzyme concentrations were still elevated. An echocardiogram prior to the patient’s discharge showed a slightly decreased ejection fraction with retrosternal hypokinesis. The patient was then discharged on warfarin, aspirin, metoprolol, and a loop diuretic.

**Table 1:** Hypercoagulable panel examination.

Test	Patient’s value	Normal range
Protein C	46%	70%-140%
Protein S	90%	70%-123%
Antithrombin III	83%	80%-120%
International normalized ratio (INR)	1.07	1
Prothrombin time (PT)	12.1 s	11-15 s
Activated partial thromboplastin time (APPT)	14.6 s	26-36 s
Factor 5 Leiden mutation	Not detected	-

## Discussion

Protein C deficiency can either be inherited or acquired, the former being passed on in an autosomal dominant fashion and its gene located on chromosome 2q13-q14. The prevalence of heterozygous protein C is 1 in 300 [6-7] and is labeled as such when protein C levels are <60%. A protein C deficiency homozygous individual has no detectable levels of protein C and unlike heterozygotes, which comes to clinical attention in the third decade of life, the homozygous state usually targets neonates as ‘purpura fulminans neonatalis.’ Protein C deficiency or resistance against its activated complex results in excess clothing factors IXa and Xa activity and hypercoagulability [6, 8]. Protein C deficiency usually leads to mainly venous disease rather than arterial with the predominant clinical symptom being recurrent venous thrombus [6]. Although the likelihood of venous disease is seven-fold that of arterial disease [9], some cases of myocardial infarctions and strokes have been reported. A study conducted in heterozygous protein C deficient patients showed prevalence of arterial disease in only 7.1% of the patients [10]. Although the number of patients who suffer from arterial thrombus on a background of protein C deficiency is low and there is not enough literature to prove the correlation between the two, it is essential to not undermine this relation. 

Management of protein C deficiency revolves around preventing thrombus formation. Lifestyle changes is an imperative part of the management and includes but is not limited to: smoking cessation, dietary changes, exercise, discontinuing estrogen supplements and contraceptives that include estrogen, and avoiding prolonged immobility [11]. The majority of protein C deficient patients do not develop thrombi and thus treatment is not required [12-13]. However, patients who are at increased risk of thrombus formation, like in the event of a surgery or pregnancy, a family history of abnormal blood clotting [14], or with previous abnormal clotting episodes are usually treated with anticoagulants such as heparin and warfarin, which lower the chance of thrombus formation in the future [12].

In 2007 the Food and Drug Administration (FDA) approved a synthetic protein C concentrate as a supplement in deficient patients. Studies have shown that high intravenous doses of synthetic protein C can help thin blood and prevent clot formation [15]. Protein C concentrate can also be used prophylactically during surgery, pregnancy delivery, or in the presence of an overwhelming blood stream infection (sepsis) [13]. As there are no guidelines as to who is to be given protein C concentrate, it is currently only being administered in high risk patients or when heparin alone cannot be safely administered due to the increased risk of bleeding [13]. However, in cases of severe protein C deficiency, protein C concentrate has been dispensed for regular use [13].

Patients with protein C deficiency are strongly advised to inform nurses and physicians about their deficiency prior to any procedure or surgery. Individuals with a family history of protein C deficiency or hereditary thrombophilia are recommended to undergo a screening process for early detection.

## Conclusions

It is imperative to acknowledge protein C deficiency as a cause of acute premature myocardial infarctions in young patients with minimal or no risk factors for cardiovascular disease as effective therapy can prevent potentially fatal consequences. Detected patients should be advised to comply with their medication and should be made aware of the possible complications of their condition. The mentioned patient will be treated with long-term warfarin, aspirin, metoprolol, loop diuretic, and lifestyle changes.
